# Greatly Improved Small Inductance Measurement Using Quartz Crystal Parasitic Capacitance Compensation

**DOI:** 10.3390/s100403954

**Published:** 2010-04-20

**Authors:** Vojko Matko, Karel Jezernik

**Affiliations:** Faculty of Electrical Engineering and Computer Science, University of Maribor, Smetanova 17, 2000 Maribor, Slovenia; E-Mail: karel.jezernik@uni-mb.si

**Keywords:** quartz crystal, magnetic pulling, parasitic capacitance compensation

## Abstract

Generally, quartz crystal inductance frequency pulling in oscillators is very low and therefore is not often used in practice. The new method of improving frequency pullability uses inductance to compensate for quartz stray capacitances. To this end, a special AT fundamental quartz crystal working near the antiresonance frequency is selected. By modifying its equivalent circuit with load inductance and series tuning capacitance, the magnetic sensing of the circuit can be highly improved. The experimental results show that the new approach using the quartz crystal stray capacitance compensation method increases the frequency pulling range (from ≅ 2 kHz/μH to ≅ 600 kHz/μH) by × 300 depending on the type of oscillator, making possible the measurement of nano-magnetic changes.

## 1. Introduction

The high precision measurement of small inductance changes has become increasingly important in sensor techniques, particularly for the measurement of nano extensions, hollow nano-sphere magnetic properties, novel magnetic nano-adsorbents, displacement field forces, *etc.* The equivalent circuits of coreless inductive elements at low frequencies contain only inductances and series resistances. At high frequencies, parallel and coupling equivalent capacitances are included. Calculation of LCR values of complicated equivalent circuits (with many LCR elements) are very tedious, so often for that purpose a special dedicated processor with appropriate software is provided in the measuring device. In metrology, complex notation is frequently used to describe linear models of inductive elements. The presented models with lumped inductance are not always a sufficiently good approximation of the real properties of electric circuits. This applies particularly to circuits made with geometrically large wires (*i.e.*, of a significant length, surface area, or volume). In such cases, the models applied use an adequately distributed inductance (linearly, over the surface, or throughout the volume) [1–2].

Impedance measurement methods for inductors are divided into three basic groups: current and voltage methods based on impedance determination, bridge and differential methods based on comparison of the voltage and currents of the measured and reference impedances until a state of balance is reached, and resonance methods based on physical connection of the measured inductor and a capacitor to create a resonant system [2].

The advantage of the current and voltage method lies in limiting the influence of stray capacitances as a result of attaching one of the terminals of the measured impedance to a point of “virtual ground”. However, to obtain small measurement errors, especially at high frequencies, amplifiers with very good dynamic properties must be used. Combined inductance measurement errors obtained by current and voltage methods depend on the following factors: voltmeter errors, errors in calculating resistance, system factors (residual and leakage inductances, resistances and capacitances), and the quality of approximation of the measured impedances by the equivalent circuit [2].

For correct measurements when using ac bridges, it is essential to minimize the influence of harmful coupling among the bridge elements and connection wires, between each other and the environment. These couplings are produced by the capacitances, inductances, and parasitic resistances of bridge elements to the environment and among themselves. The joint error of the inductance measurement results depends then on: the accuracy of the standards used as the bridge elements, mainly standard resistors and capacitors; errors in determining the frequency of the bridge supplying voltage; errors of the resolution of the zero detection systems; errors caused by the influence of residual and stray inductances; resistances and capacitances of the bridge elements and wiring; and the quality of approximation of the measured impedances in the equivalent circuit [2].

Resonance methods are a third type of inductance measurement method. They are based on the application of a series or parallel resonance LC circuit as elements of either a bridge circuit or a two-port (four-terminal) “T”-type network. The joint error of the inductance measurement results are similar as when using ac bridge method (depending on the quality of the elements) [3].

Instruments commonly used for inductance measurements are built as universal and multifunctional devices. The results of inductance measurements depend on how the measurements are preformed, including how the measured inductor is connected to the meter. Care should be taken to limit such influences as inductive and capacitive coupling of the inductive element to the environment, sources of distortion, and the choice of operating frequency (Hewlett-Packard 4284A—Precision LCR meter, 0.01 nH–99.9999 kH, 20 Hz–1 MHz, 0.05%) [2].

The quartz crystal method is another possible method. A prior study of the quartz crystal pulling increase has shown that it is also possible to increase the inductive frequency pulling [4]. The latter is usually a lot smaller than the capacitive one and is as such not often used in practice. However, the great advantage of this method is smaller parasitic capacitance, inductance and resistance, on the one hand, and high sensitivity and stability of small inductance change measurements, on the other. The usual inductance pulling is in the range up to 2 kHz/μH, but through an appropriate compensation of the stray capacitance of the housing and the crystal element (which is a novelty in this research), inductance pulling can be greatly improved. This research focuses on the inductance pulling sensitivity of the AT fundamental quartz crystals operating over the measurement temperature range of 10°C to 40 °C. Crystals fabricated in this manner exhibit excellent frequency *versus* temperature stability. They have fundamental resonant frequencies between 3.5 and 10 MHz [4].

## 2. Inductance Compensation of Quartz Parasitic Capacitance

**Figure 1.**
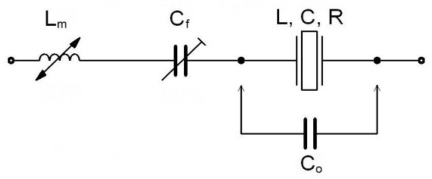
Compensation inductance *L_m_* (sensing inductance) and fine tuning capacitance *C_f_* in series with the quartz crystal.

In the equivalent circuit of an electrical representation of the quartz crystal’s parasitic capacitance *C_o_* represents the capacitance of the crystal element and holder (Figure 1). Oscillator crystals are normally designed with *C_o_* less than 5 pF. One possible way of increasing the inductance pulling sensitivity is to compensate *C_o_* using an inductance *L_m_*. By decreasing the values of *k* = (1, 0.5, 0.33), *C_o_* in (1/*k*) · *C_o_* increases, changing the value of *L_m_* into *k* · *L_m_* (Equation 1) [4–10]:

(1) $$L_{m}=\frac{1}{\omega_{0}^{2}\cdot \left(\frac{1}{k}\right)\cdot C_{0}}$$

The special value of the chosen compensated *L_m_* (sensing very small magnetic changes) due to high sensitivity represents a novelty in this research. Due to the small inductance *L_m_*, the real resistance *R_m_* can be ignored. The series capacitance *C_f_* is using for fine (quartz resonsant) frequency tuning. The components *L*, *C*, and *R* represent the mechanical behavior of the crystal element [4,6].

**Figure 2.**
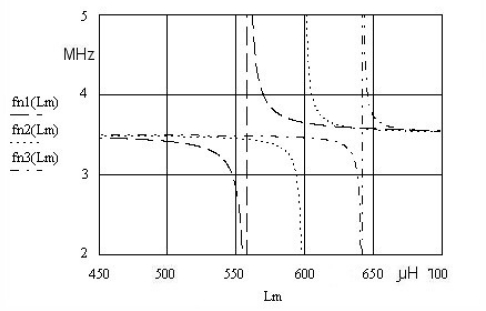
Frequency pulling for fn1 (*L_m_*) for *k* = 1, fn2 (*L_m_*) for *k* = 0.5 and fn3 (*L_m_*) for *k* = 0.33 in relation to the change of *L_m_*.

Using the compensation criterion (Equation 1) we get Equation 2, which represents a new series resonant frequency for the circuit (Figure 1). The quartz series resonant frequency without *L_m_* is represented with *ω_0_*. Depending on the selected value of *k* (*k* = 1, 0.5, 0.33), the value of *C_o_* changes producing three different resonance curves (Figure 2), for the *L_m_* inductance range (450–750 μH). A more detailed theoretical background explaining Equation 2 is provided in references [4] and [6].

(2) $$\mathrm{fnk}\left(L_{m}\right)=\frac{1+\frac{C}{2\left(\frac{1}{k}\cdot C_{0}-\frac{1}{\omega_{0}^{2}\cdot k\cdot L_{m}-\frac{1}{C_{f}}}\right)}}{2\cdot \pi \cdot \sqrt{L\cdot C}}$$

Equation 2 contains the criterion (Equation 1) with a factor *k* determining the value of *L_m_* in relation to the given *C_0_*.

## 3. Experimental Results

The experimental data values in the quartz crystal equivalent circuit were measured by a HP 4194A impedance/gain-phase analyzer. The quartz crystal (in a HC-49/U housing) was selected due to its high Q value (Table 1) and consequently, its stable oscillation [4]

The frequency *f_r_* represents a fundamental quartz crystal resonant frequency (Table 1). The value *k* (Equation 2) determines the ratio between the compensation inductance *L_m_* and *C_0_*. Figure 2 shows Equation 2 for three different *k* values (*k* = 1, 0.5, 0.33) at the constant value of *C_f_* = 50 pF.

**Figure 3.**
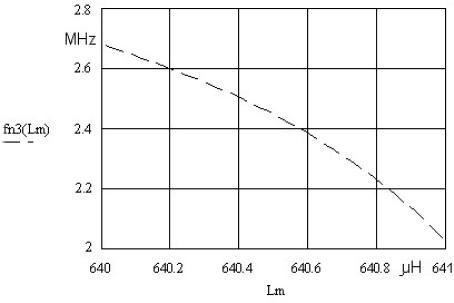
Frequency pulling fn3 (*L_m_*) for *k* = 0.33.

In the case of fn3 (*L_m_*), where *k* = 0.33 and *L_m_* = 640 μH, the most steep change of the resonant frequency close to quartz crystal antiresonance frequency can be observed. For the range of inductance change 1μH at *L_m_* = 640 μH the oscillator frequency change is approximately 600 kHz (Figure 2 and Figure 3). The change is non-linear, but with an appropriate approximation method it can be linearized. To get a steeper change, *C_0_* should be < 1 pF, which practically does not exist in the currently manufactured crystals.

**Figure 4.**
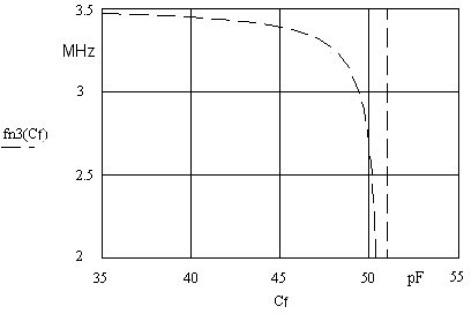
Fine-tuning of the working frequency using *C_f_* (35 up to 55 pF) at constant value *L_m_* = 640 μH.

The capacitance *C_f_* in the circuit (Figure 1) is used to set the frequency working point at a given chosen inductance (Figure 4). By setting the inductance working point, greater or smaller frequency sensitivity can be achieved. The capacitor *C_f_* must be of high-quality (made of ceramics).

## 4. Frequency Sensitivity and Stability

Experimental results show the quartz crystal frequency sensitivity in relation to the applied use of circuits and increased inductance frequency pulling. Taking into account the long-term quartz crystal frequency stability (0.1 Hz) (described in reference [5]), the frequency pulling increase (at the frequency change of 600 kHz/1μH) (Figure 3) gives the lowest inductance change 100 fH/0.1Hz. If, however, the short-term (one day) crystal frequency stability (0.01Hz) is taken into account, the lowest inductance change is 10 fH/0.01Hz [1–5].

The most common factors affecting frequency stability such as operating temperature range, aging, hysteresis and drive level as well as all other crystal characteristics influencing the stability should also be considered because a stable oscillator circuit plays an important role in the increase of pulling and linear frequency dependence. As a consequence of hysteresis, the frequency *versus* temperature curves obtained by slowly increasing the temperature from, say, 10 °C to 40 °C will not coincide with the curve obtained by slowly decreasing the temperature from 40 °C to 10 °C. Frequency stability also depends on the temperature coefficient of the inductance material used of *L_m_*. In general, the oscillator’s circuit long-term stability also depends upon the crystal aging. Cold weld packages which are specially processed and welded under a high vacuum offer better aging rates and typically the aging rates of cold weld crystals is less than ±1 ppm/year (10 °C to 40 °C). Stability of the electronic circuit depends upon the circuit type and quality of its elements. Also, it is important that the drive level in the oscillator application not exceed 30 μW [1,5,11–14].

## 5. Conclusions

Experimental results show that the use of series inductance compensated crystals with a special determined value of *L_m_* increases the pulling range by ×300 for *k* = 0.33, depending on the circuit used. This high pulling increase represents a novelty and a major advantage of the method discussed in the measurement of femto-Henry ranges. With fine tuning a series load capacitance connected in series with the crystal, the frequency of the oscillator is set to an appropriate output circuit frequency. It should also be emphasized that the exact pulling limits depend on the crystal’s Q value as well as associated stray capacitances and the factor *k*. The inductance *L_m_* is determined from known stray capacitances and the known factor *k*. Given the higher Q value, the frequency range below 10 MHz is very favorable because it influences high quartz oscillation stability. The increased pulling range obtained experimentally can be used for determination of many different measurements such as nano inductance, nano-extension and compression, nano-positioning, angle, pressure, nano-force and other non-electrical quantities [15–17].

## Figures and Tables

**Table 1.** Quartz data for resonant frequency 3.5 MHz [4].

***f_r_* (MHz)**	***R*(Ohm)**	***C*(fF)**	***L*(mH)**	***C_o_*(pF)**	***L_m_*(uH)**	***Q***
3.5	10	25	82.83	4	520.0	181980
